# “It’s Like Jogging Next to the Highway”: A Qualitative Analysis of the Motivations and Experiences of Single-, Dual-, and Ex-Users of IQOS in The Netherlands

**DOI:** 10.3390/toxics10060283

**Published:** 2022-05-26

**Authors:** Anne Havermans, Lotte E. van Nierop, Charlotte G. G. M. Pauwels, Reinskje Talhout

**Affiliations:** Centre for Health Protection, National Institute for Public Health and the Environment (RIVM), P.O. Box 1, 3720 BA Bilthoven, The Netherlands; lotte.van.nierop@rivm.nl (L.E.v.N.); charlotte.pauwels@rivm.nl (C.G.G.M.P.); reinskje.talhout@rivm.nl (R.T.)

**Keywords:** heated tobacco products, qualitative research, focus group, appeal, experience, motivation, (risk) perceptions

## Abstract

The popularity of heated tobacco products (HTPs) is of concern, as most users are dual users exposed to emissions of both HTPs and conventional cigarettes. Furthermore, HTPs may appeal to young people and non-smokers. This study aims to build intelligence on user experiences in order to inform policy development. We conducted five semi-structured focus group interviews with single-, dual-, and ex-users of the HTP IQOS. The discussions focused on initiation and use, experiences and perception, and knowledge and information needs. We performed a thematic analysis of the transcripts. All users smoked cigarettes and/or roll your own (RYO) tobacco before using HTP. We found that almost all users started using IQOS after being introduced to it by others. Single users successfully quit smoking cigarettes using the IQOS, liked the taste, and experienced physical benefits. Dual users experienced more satisfaction from smoking cigarettes and used the IQOS for specific occasions, such as social situations or in places with smoking bans. All IQOS users described themselves as smokers and considered using the IQOS as an alternative way of smoking. Regulators may consider providing reliable and easily accessible information and regulating points of sale, promotional activities, and product properties such as flavors and devices in order to reduce product attractiveness and discourage use.

## 1. Introduction

Heated tobacco products (HTPs) use battery-powered devices to heat sticks consisting of compressed tobacco and additives. They are typically marketed as “less harmful” alternatives to smoking conventional cigarettes in many countries. Since 2014, various new HTPs have been introduced, including Philip Morris’ IQOS [1,2]. In Europe, use prevalence has been relatively low (e.g., 0.1% current users in 2016–2018) [3], but appears to be increasing. In 2020, on average, 6.5% of participants in the Eurobarometer study had ever used an HTP, 1.3% were current users, and 0.7% daily users [4]. In the Netherlands specifically, ever use was reported at 3–4% and current use at 0.4–0.5% in 2020 [4,5]. This increasing popularity of HTPs is concerning for several reasons. First, even though most HTP users are current or former smokers [4,6], the majority of them (75–96%) are dual users of both HTPs and conventional cigarettes, and therefore are exposed to emissions from HTPs as well as conventional cigarettes [7,8,9,10,11]. Furthermore, HTPs have at least some appeal to youth and young adults, including non-smokers [12,13]. Data suggest that IQOS may attract non-tobacco-using adolescents and young adults to initiate tobacco use [1,14].

In order to understand and act upon the increasing popularity of these products, it is important to know which factors make HTPs attractive to use. In that respect, marketing, product image, and product features are important factors and a good starting point to understand and predict actual use. HTPs are marketed as “reduced-risk”, “alternative,” “clean,” “smoke-free,” “innovative,” “chic,” and “pure”, and “helpful to smokers who want to quit” [2,7,15,16,17,18]. IQOS stores are often prominently and strategically situated in high-end areas in selected cities [1,19,20,21,22], and the IQOS name, device, packaging, and stores resemble those of popular cell phone brands that attract young people [1,23]. Overall, HTP’s image is rather different from the image of conventional cigarettes: a high-demand, upscale product for people interested in new technologies [1]. The heat-stick packages are sleek, with pack colors indicating flavor type and strength, as plain packaging and graphic warning labels are not required in most countries [18]. The availability of non-tobacco flavors [24,25] is especially attractive in countries with flavor bans on cigarettes, such European countries and the US [26,27].

However, marketing strategy and product image data are not sufficient to understand actual use. Thus, data on consumer attitudes, experience, and perceptions are crucial. For example, a recent study showed that the sleek electronic design and lower perceived harmfulness of IQOS appeal to young adult smokers [23]. Among smokers who have used them, HTPs are generally not considered as sensory and psychologically satisfactory as cigarettes [28,29,30,31,32]. Rather, the most often-cited reasons to use were the belief that HTPs are “less harmful” or may support conventional cigarette smoking cessation [4,5,11,33,34,35,36,37,38,39]. For those who continue using them, liking of the smell, taste, aftertaste, and ease of use are important predictors of continuation [40]. Perceived sensory benefits reported by users are less throat discomfort, better smell, appealing packaging, cleanliness, lack of ash and smoke, and availability of flavors [5,16,39]. Other reported reasons to use were lower costs, satisfaction and enjoyment, use in smoke-free places, new technology, and the product being more socially acceptable [5,11,37,38,39]. Disadvantages were issues of accessibility, and shortcomings with maintenance/operation limiting ongoing use [37].

While several studies are available on marketing, product image, product features and perceptions, and reasons to use HTPs, many data gaps exist regarding user experience. In particular, qualitative research data describing user experiences, attitudes, motivations, knowledge, and risk perception are scarce. The majority of the existing data come from studies where participants select their answers from predefined multiple choice answering options. This leaves little room for spontaneous thoughts and to discover new or unexpected motivations and experiences. Moreover, when survey studies base their questions and answering options on previous literature that (mainly) consists of more surveys, confirmation bias may arise.

So far, only three studies used a qualitative approach to obtain an elaborated account of HTP users’ experiences. These include focus groups conducted in South Korea [41], the United Kingdom (UK) [37], Japan, and Switzerland [16]. Besides cultural differences, these countries also have very different tobacco product landscapes and tobacco control efforts between them and compared to most Western and European countries. For example, after its introduction to the market, the prevalence of e-cigarette use among adolescents in South Korea remained very low (4%), as opposed to increasing numbers in other countries [42]. Both Japan [43] and Switzerland [44] have banned the sales of nicotine-containing e-cigarettes, while only Japan has a low prevalence rate among young people (5%, compared to 24% in Switzerland). The UK, on the other hand, is known for its positive attitude towards claimed “reduced-harm” products, including e-cigarettes [45], but also has a relatively low prevalence of e-cigarette use among young adults (3–6%) [46]. These local differences in regulation and attitudes towards e-cigarettes may also trigger diverse motivations and perceptions regarding HTP use. Moreover, different flavors of IQOS heat-sticks (HEETS) are available in different countries. For example, in South Korea and Japan, various sweet flavors are sold that are not available in other countries, such as citrus and tropical menthol, and in the UK, Sienna HEETS with menthol capsules are available [47]. Thus, it is important to investigate user experiences around the world, not just to inform local policy makers, but also to obtain a better understanding of global similarities and differences, and the effects of tobacco regulation. Therefore, the current focus group study explores motivations, experiences, perceptions, and knowledge among Dutch single users, dual users, and former users of HTPs.

## 2. Materials and Methods

To explore experiences and perceptions regarding HTP use, we conducted five online focus groups: one group of single users, two of dual users, and two of ex-users. Research agency Kantar (Amsterdam, the Netherlands) approached members of their panel who had previously indicated ever using an HTP (*n* = 1301). A total of 57 HTP-ever-users expressed interest in participating in the current study, and filled out a brief questionnaire regarding their product use and demographic characteristics. They were allocated to focus groups based on their user status (ex-, dual-, or single users), aiming for heterogeneous groups with respect to age, gender and level of education. Participants were invited via email and were contacted by phone a few days before the focus groups took place, to ensure participation. Finally, 19 HTP users participated in the focus groups: one group of five single-users, two groups of respectively two and three dual-users, and two groups of respectively four and five ex-users.

The focus group sessions were conducted online via Webex Meetings (Webex.com, accessed between 12 and 20 July 2021). The sessions had to take place online due to ongoing COVID-19 restrictions at the time. Informed consent was obtained before the session by email and verbally, and the participants received a EUR 30 gift card incentive after participation. The discussions were guided and monitored by two of the authors (A.H. and C.G.G.M., respectively), following a semi-structured protocol based on topics, open-ended questions, and prompts (Table S1) [48]. The topic list was developed based on published qualitative studies among HTP and e-cigarette users, supplemented with the authors’ expert knowledge and tested and optimized in a pilot discussion with a current HTP single user.

The focus group session started with a general introduction of the study and proceeded with discussions around three central themes, i.e., initiation and use, experiences and perception, and knowledge and information needs (see Table S1). For the second theme, the participants used an interactive online discussion board (Mural) to visualize their thoughts. The focus groups lasted from one hour and 20 min up to two hours, depending on the number of participants (i.e., focus groups with more participants were longer.) All sessions were audiovisual recorded.

The focus group sessions were transcribed verbatim and analyzed using a thematic analysis approach [49]. Two of the authors (A.H. and L.E.v.N.) performed open coding on one transcript each, using Atlas.ti. 9. Codes were generated and collated based on thematic similarity. For example, text with codes related to taste or feel were grouped into the overarching theme “sensory experience”. The two resulting sets of codes and potential themes were compared, discussed, and refined. These steps were repeated for the other three transcripts. Finally, the themes and subthemes were reviewed and defined in consultation with a third author (C.G.G.M.P.).

## 3. Results

### 3.1. Participant Characteristics

All participants used the IQOS device with HEETS heat-sticks. They all smoked cigarettes or roll your own (RYO) tobacco prior to initiating IQOS use. An overview of participant and use characteristics is provided in Table 1.

### 3.2. Initiation and Use

#### 3.2.1. First Contact HTP

Most participants indicated that their first experience with the IQOS was through a family member, friend, or colleague who was already using it. This way, they were informed about the product and, in some cases, could try it. Others indicated that they were informed about IQOS by a salesperson or customer in a place where they would buy cigarettes, such as a tobacconist shop or petrol station, and in one case, a café. Other ways in which people became familiar with the IQOS were by participating in research or consumer panels, receiving it as a gift, or searching for an alternative to conventional smoking.

#### 3.2.2. Reasons to Start Using IQOS

The most frequently mentioned reason for starting to use IQOS was the belief that it could be a “healthier alternative” to regular smoking. Aspects mentioned in relation to this belief were lower amounts of tar and nicotine in the product. Participants further mentioned wanting to reduce or quit smoking regular cigarettes with use of IQOS. Another reason mentioned for using IQOS is that its use was allowed in places where smoking is not permitted (Before 2020, heated tobacco products were not included in the Dutch smoking ban). Participants indicated that the IQOS is a practical alternative that enables you to smoke indoors without bothering others or get smelly hands or clothing. One single user explained: *“I had been arguing with my wife quite often about the stench and I had to smoke outside and it was all very difficult … but now with the IQOS, I can smoke inside again”*. A number of participants were ex-menthol cigarette smokers who started using IQOS as a replacement when these were banned. They considered the IQOS (with menthol flavored HEETS) as the best substitute, compared to menthol-flavored e-cigarettes and waterpipes. Finally, some ex- and dual users indicated that they wanted to try the IQOS out of curiosity, as it looked attractive and interesting. For example, they wondered whether it would equal the satisfaction of conventional smoking, have any advantages over conventional smoking, or whether it would be convenient to use at certain times (e.g., lunch breaks). Generally, they were looking for something that would provide the same satisfaction as smoking, but was cheaper and/or less unhealthy.

#### 3.2.3. Moments of Use

Most former and dual users indicated that they used the IQOS at specific moments. Some chose to use it when they were in the company of others and did not want to bother them, for instance, at work or with friends. Others indicated that they preferred to smoke regular cigarettes when in social situations and use the IQOS when they were alone, for example, to quickly meet their nicotine needs. Smoking a cigarette was also more often described as a “reward” or “moment for yourself”. One dual user described this as *“With the IQOS, it’s often a quick stop taking a puff or two; with a cigarette, you really take your time. It is like eating at home when you eat faster than when in a restaurant where you really enjoy your meal, look at the tables and people around you, you enjoy everything more”*. However, for another participant, using the IQOS created a “moment of pleasure”. Some dual users alternated the use of cigarettes and the IQOS when they felt like doing something different or experiencing a different flavor. These were mainly participants who used menthol-flavored HEETS. Some also considered the IQOS to be practical for use in the car, as there is no ash and cigarette butt to get rid of. Some ex- and single users use(d) the IQOS as a complete replacement for cigarettes. Several single users mentioned a decline in IQOS use over time, which was intentional for some and not for others. Some dual users indicated that their cigarette smoking habits had not changed (i.e., they did not smoke less) since they started using IQOS occasionally.

#### 3.2.4. HEETS Flavors

All participants indicated that they stuck to using one HEETS flavor; most of them used a tobacco flavor that was close to the cigarettes that they were used to, while some preferred a menthol flavor for the same reason. Relatively speaking, many single users used menthol flavored HEETS. Most stuck with the same flavor from the start, and did not seem very interested in the variety of flavors available. A few indicated that it is too expensive to try multiple flavors, because when a flavor is not pleasant, the whole package of HEETS becomes useless. Some indicated that they had tried different flavors to find out which one is their favorite, but were not really impressed with most of the flavors.

#### 3.2.5. Reasons to Quit IQOS

Ex-users reported quitting IQOS because they disliked the taste or it did not relieve their nicotine craving. Many mentioned a strange, bitter, and/or bad aftertaste. They also experienced that the overall taste and/or “feeling” was not the same as that of cigarettes or RYO tobacco, which was something they could not get used to. Two ex-users intended to reduce their nicotine intake by using the IQOS and expected it to contain no or hardly any nicotine. They quit when they found out that it did: *“I thought IQOS contained less nicotine, but then it turned out that it contained the same amount. Then I will not deprive myself of the good taste of RYO”*. One ex-user quit because she experienced a bad headache each time she used the IQOS, which she never experienced when smoking. For some ex-users, the high costs (particularly relative to RYO tobacco use) also played a role in their decision to quit. One dual user indicated that he was motivated to quit using the IQOS for that reason, while another dual user indicated that he intended to continue using it regardless. Some single users indicated that they would not return to smoking, even if the IQOS would be no longer available.

### 3.3. Experiences and Perception of HTP

#### 3.3.1. General (First) Impression

Ex- and dual users described the IQOS as a fancy, new, and interesting gadget to try out. However, after using it, they were unimpressed, as the IQOS did not meet their expectations; it was nothing special to them and did not have a “wow” effect. A former user mentioned: *“It does not taste the same as a cigarette and I could not get used to it, I didn’t like it, I’d rather go for the real thing”*. They also found the IQOS expensive and inconvenient to use, e.g., that it switched off after a short time and does not show when a HEET is finished. It was also noted that to people unfamiliar with the product, it could seem that IQOS users were using drugs (i.e., it looks like vaporizing cannabis). Single users were more positive about the IQOS and described it as tasty and less bothersome to others. They also mentioned a noticeable improvement in physical symptoms, such as less coughing and no more yellow nails. One user noted that finding a preferred flavor is challenging, as the taste descriptions of the HEETS (e.g., creamy, full, malty, and spicy) are not relatable to the taste of cigarettes.

#### 3.3.2. Physical Effects

Most single users experienced positive effects of IQOS use on their body and health compared to smoking cigarettes/RYO tobacco. They felt fitter, less out of breath, and coughed less. Some also described that their taste had improved; for example, one person mentioned that she could better recognize the spices in a dish. A person who used to have yellow teeth and fingers due to smoking said that these disappeared with use of the IQOS.

#### 3.3.3. Sensory Experience and Rituals

##### Overall

Many dual and former users indicated that the sensory experience of the IQOS did not come close to that of a conventional cigarette. This is due to its taste and nicotine strength (as described below) and the overall sensory experience. For example, a dual user explained: *“Well, you do notice nicotine, but I found that you felt much less like smoke or something like that actually getting into your lungs, it felt much lighter and therefore much less satisfying”*. Some dual users indicated that they did not experience the same pleasure from the IQOS as from a cigarette. Besides the sensory experience, this also had to do with the actions of using it. When smoking, there was often a ritual (opening the package, smelling the cigarette, lighting it) that was experienced as pleasant. They also explained that they purposefully create some time to enjoy their cigarette at certain times, for example after getting up in the morning. The use of the IQOS was described more as something “additional”, or for use in between other activities. Using the IQOS did not cause as much of an “enjoyable moment” as a real cigarette. For example, one dual user explained: *“I just don’t find the pleasure of smoking the IQOS, that pleasure that you have when lighting a cigarette. But it’s nice to have on the side”*. Some dual and single users found the IQOS just as satisfying as a cigarette. One of them had been using the IQOS for so long that he could no longer make a good comparison. It was also mentioned that the IQOS, of all replacement products (e.g., e-cigarette, IQOS, waterpipe), is the closest to a cigarette.

##### Taste

All ex- and most dual users did not like the taste of the IQOS (HEETS). The taste was different from that of a conventional cigarette and most had to get used to it. It was described as “chemical” or the taste of cheap (foreign) tobacco or cigarettes. One ex-user said: *“I really liked that taste much less than normal cigarettes. Really like some cheap tobacco, or very cheap cigarettes that you can buy abroad. No, I thought it was really an inferior taste”*. Some reported experiencing a bad aftertaste (like an ashtray) that lingered for a long time and that was not experienced after smoking cigarettes or RYO. The bad taste was seen as an advantage by one ex-user, because it allowed him to reduce his IQOS use in a short period of time. Some dual and all single users thought the taste was fine, and some single users even liked it better than a cigarette.

##### Nicotine Buzz

Many dual and former users indicated that the IQOS did not match the intensity and kick of a cigarette and was less satisfying. Some mentioned that they did notice the nicotine, but it was not enough to fulfill their needs. Some considered it a disadvantage that the nicotine strength could not be adjusted with the IQOS, as opposed to e-cigarettes that can be used to titrate and reduce nicotine intake.

#### 3.3.4. Smoke and Smell

All user groups found it an advantage that less side stream smoke is emitted when using the IQOS. This was beneficial for themselves, for example, because they no longer smelled bad, but more importantly, they liked that they were no longer a burden to others. Moreover, the use of the IQOS was described as more discreet than the use of e-cigarettes, which can produce large vapor clouds. Several single users mentioned that people around them were still bothered by the smell of the IQOS, indicating that there are differences between the experiences of people. They described a sewage smell for a brief moment after starting to use the IQOS. A few people also mentioned that the IQOS smelled worse than e-cigarettes, according to themselves and people in their close surroundings, which can be considered a disadvantage.

#### 3.3.5. Device

Participants from all user groups described the IQOS device as attractively designed and modern. Words like fancy, trendy, hip, special, and exotic were mentioned in the different user groups. The participants considered the design of the product to be well-thought-out with regard to its appeal, but less so with regard to the ease of use (see Section 3.3.6). The store and website were also described as sleek-looking by those who had visited them. The single users were the most enthusiastic about the design and showed and described different models and accessories to others in the group. Users from other groups more often indicated that the design was not important to them. Many users mentioned the compact size as an advantage, especially compared to certain e-cigarette models. Yet, some described the IQOS as large and bulky and considered the size as negative; it comes across as showy and is not as comfortable to hold between the fingers as a cigarette or RYO.

#### 3.3.6. Ease of Use

Many former and dual users mentioned that using the IQOS was a lot of hassle. This was mainly due to the handling of the device and the fact that it had to be cleaned and charged regularly. For example, one ex-user mentions: *“But I have to say, I really found it a bit of a hassle. Put that cigarette in that device, you have to clean that device every now and then. I’m at work on the tenth floor, it’s quite a journey to go down where I can smoke. So, I sometimes smoke two cigarettes in a row. You have to recharge that device, I thought it was a hassle”*. According to many users, the battery of the IQOS ran out quickly and it was bothersome that they could not use it when it did. This was a particular disadvantage in comparison to e-cigarettes with longer-lasting batteries. Some users found a routine in charging and therefore did not consider it a problem. When using the IQOS, users typically consumed a whole HEET, after which the IQOS automatically switched off. This is considered less convenient compared to e-cigarettes that can be consumed at any time and with any number of puffs. Furthermore, several people found it impractical that it was not clearly visible when the HEET is used up, unlike an actual cigarette. The IQOS can also switch off before the HEET is used up. Users considered this a waste of the HEET, as, in their experience, it could no longer be used afterwards. One single user perceived the switching off of the device as an advantage, as the deliberate break between HEETS triggered a sense of craving for the next one and thus kept them addicted. For them, this made the IQOS a suitable replacement for cigarettes. Some users found it inconvenient that the HEETS were not for sale in many places. Users also did not find the device easy to carry, and for many dual users, it was not part of their routine to take it with them. For example, one dual user explains: *“It’s also just habit. You just put cigarettes in your pocket or in your jacket pocket. With the IQOS, I hardly ever do that, almost never. It’s really like we said before, I really have to think about that to take with me. It’s not like a roll-your-own or cigarettes. I don’t really forget those”*. Some users found the IQOS easy to use compared to cigarettes or rolling tobacco that you still have to light or roll. Several users considered it an advantage that the IQOS was easier to use in the car, because it did not produce any ash.

#### 3.3.7. Costs of Using IQOS

Many users and former users found the use of the IQOS (too) expensive, mainly because of the high costs of the HEETS. This was especially true for (former) RYO smokers. Compared to cigarettes, using the IQOS is cheaper, which was an advantage for some. However, most found the IQOS less worth the money than cigarettes. For example, one dual user said: *“Yes, you normally do that with other products. What you enjoy more, you will pay more for. What you don’t really enjoy, you pay a lot less for, or you don’t buy it at all”*. For some participants, cost was not a factor in their considerations about using the IQOS.

#### 3.3.8. Self-Perceptions of IQOS Users

IQOS users described themselves as “smokers” and generally referred to the use of the HTP as “smoking (a cigarette)”. They did this mostly out of habit or to avoid having to explain to others. They also considered the HEETS to be a specific type of cigarette. One person explained they “smoke cigarettes which [they] insert into the IQOS”. Some also indicated that they feel more targeted by media messages regarding “smokers” than “HTP/IQOS users”. All user groups indicated that others often reacted curiously and asked questions when they were using the IQOS. Some even asked to try it out. Some users have received skeptical reactions from others, for example, from those who wondered whether this is really healthier than smoking. Some also thought that IQOS use can come across as weird or “showy” to others. Several participants had the feeling that they were still seen as smokers by others, with (in their words) the negative reputation that comes with that. Some indicated that they received fewer reactions from others to the use of the IQOS than to smoking. They thought that the IQOS was more accepted than smoking, because others were not bothered by smoke and because its use was allowed in places where smoking is not allowed.

### 3.4. Knowledge and Information Needs

A number of participants indicated that they searched for information about the IQOS online before purchasing one. This concerned practical information, such as product features and instructions for use, which was found on the IQOS website and web shops. Some were informed about this in the store. None of the participants searched for information about the health consequences of IQOS use, but all of them seemed very interested in learning more about it during the course of the discussion. Most believed that HTP use is unhealthy and wanted to know whether it is really less harmful than smoking conventional cigarettes. Some were skeptical about this, or even felt misled by the manufacturer’s implications of reduced harm. For example, a dual user mentioned: *“But it doesn’t make me healthy, no. I always say: it’s a bit like jogging along the highway. You think you’re doing something healthy, but actually…”*.

The interviews also revealed a number of misconceptions about the IQOS among the participants—for example, that it did not contain nicotine or tar. One single user mentioned not knowing whether there is nicotine in it. During the conversations, information was shared between the participants and in some cases searched for online, which turned out to be eye-opening for some. For example, after a quick, spontaneous online search, an ex-user said: *“I really didn’t know that you also got tar and everything in there”*. When asked, various participants indicated that they believed that providing clear information about the harmfulness of the IQOS (in relation to cigarettes) is the responsibility of the government, and that they did not trust the information provided by cigarette manufacturers.

## 4. Discussion

In this study, we explored the experiences of single-, dual-, and ex-users of HTPs in the Netherlands. All participants used the IQOS device with HEETS heat-sticks and were prior smokers of conventional cigarettes or RYO tobacco.

### 4.1. Initiation and Use

Almost all participants started using the IQOS after they were introduced to it by someone they know, or through promotional activities from sellers. By seeing someone else using the product and speaking positively about it, their interest was triggered and they wanted to try it out for themselves. Most participants wanted to use the IQOS based on the assumption that it could be a “healthier alternative” to smoking or it would help them to reduce or quit smoking. Other reasons mentioned are social acceptability, to avoid smoking bans, out of curiosity, and as an alternative to menthol cigarettes (which are banned in the EU). These reasons for use are in line with several previous studies among HTP users [4,11,33,41]. Additional identified reasons for use from other studies are use by friends or family, enjoyableness, lower costs, and nice flavors [4,5,37,39]. While these were not the main reasons for use in our study, they did come up when further discussing initiation and benefits of the IQOS. Only a few users came across the IQOS on their own initiative, because they were looking for an alternative to conventional smoking. Together with the low prevalence rates, this suggests that IQOS may not be a commonly searched-for smoking cessation product.

Our findings shed more light on some of the commonly indicated reasons for use in questionnaires. For instance, several studies reported use by family and friends as a reason for use. In our study, we found that several participants became familiar with the IQOS through someone they know, and this seems to have led them to initiate use. However, the actual reasons or motivations for use that were mentioned had nothing to do with friends or family using it, but were related to, for example, potential health benefits or social acceptability. Therefore, we suggest clearly defining and distinguishing causes (i.e., the factor that triggers initiation) and reasons or motivations (i.e., why they want to use it) for use in future research in order to improve interpretation of the findings. Curiosity is also a frequently indicated reason for the use of tobacco products such as HTPs [5]. However, this answer is not very informative, as it seems obvious that curiosity precedes the adoption of a new product and it does not reveal any information about the underlying motivations and/or beliefs. Our qualitative study design allowed for a deeper questioning about the motives behind curiosity. That is, participants explained that they were curious about the IQOS because of its attractive and interesting design, and were interested to know how it compared to smoking in terms of satisfaction and potential advantages. Some participants wondered whether the IQOS would be a good solution to use at times when smoking was considered inconvenient (e.g., around other people, or in smoke-free places). Our findings show that curiosity, as a reason by itself, is not sufficiently informative to understand people’s true motivations for initiating product use. We recommend future studies to take this into account, for example when developing user surveys.

### 4.2. User Experiences

Our study showed that ex- and dual users expressed an overall moderately negative impression of the IQOS. It did not live up to their expectations of finding an alternative product with a comparable experience to cigarettes or RYO tobacco. The latter two were often associated with a behavioral ritual that contributes to an overall enjoyable moment, which they found lacking when using the IQOS. Interestingly, actions surrounding smoking were considered rituals that increase the pleasurable experience, while actions surrounding IQOS use were considered bothersome by ex- and dual users. This may be due to the fact that they were less habituated to the IQOS than to conventional cigarettes. Single users also did not describe a pleasurable ritual related to IQOS use, but did not experience practical inconveniences of the IQOS either, potentially due to being more habituated to its use. Overall, our participants experienced the actions of using the IQOS as being different from smoking. In contrast, a study with IQOS users in the UK found that users felt encouraged to use the IQOS, as it allowed them to maintain the same rituals and routines as when smoking cigarettes [37].

In line with our findings, several other studies also showed that HTPs are considered less satisfying and pleasant-tasting than cigarettes [28,29,30,31,32,41]. However, in one study, a large percentage (72.0%) of ever HTP users found them at least as satisfying as smoking [39]. As expected, perceived satisfaction relative to smoking was higher among current users of HTPs than among those who had tried a few times or were using HTPs less than monthly. In our study, several single users also indicated equal satisfaction from IQOS and cigarette smoking. Thus, it seems that users who are more habituated experience more satisfaction from using the IQOS. However, it is also possible that users who experience more satisfaction use IQOS more, and become single users. In our study, single users were also noticeably more positive about the IQOS; they experienced more physical benefits, liked the taste, were more satisfied, and valued the design of the device more. Most single users used menthol-flavored HEETS and some of them started using the IQOS as a replacement for menthol cigarettes.

The availability of different flavors of HEETS was not considered an attractive aspect by most users in our study. Participants generally chose a flavor that was most similar to the cigarettes or RYO tobacco they were using, and stuck with this flavor. Our findings are not in line with two previous studies that found flavors to be one of the main reasons for using HTP [5,39]. However, the availability of flavors as a reason for use indicated in a questionnaire is open to multiple interpretations, as this could point to the availability of different flavors allowing someone to alternate use, or it could point to the availability of a certain flavor they like (among others). Our results show that it is not necessarily the choice from several options that is attractive, but to have an option that suits your preference. Moreover, menthol is clearly a preferred option to many, and menthol IQOS users seem to be more likely to be single users. It should be noted that the flavor availability and variability of HTPs differs between countries. For example, in the Netherlands, only tobacco-like and menthol flavors are currently sold, while in other countries, various sweet flavors and HEETS with flavor capsules are marketed [50]. Therefore, it is important to compare data from user studies in multiple countries to better understand the role of flavors and flavor regulation in HTP use.

Our participants described several advantages of IQOS use. Most commonly mentioned were the experienced health benefits, being less bothersome to other people (i.e., less smell and smoke), and its stylish and convenient design. The focus group participants in Japan and Switzerland mentioned the same benefits [16], in addition to less throat discomfort and more appealing packaging. Our participants generally disliked the taste, cost (particularly in comparison to RYO tobacco), and practical inconvenience of using IQOS, which is also mostly in line with the experiences of the Japanese and Swiss focus group participants [16].

The IQOS users in our study, including single users, described themselves as smokers. They viewed the IQOS as a different type of cigarette, referred to the action of IQOS use as smoking, and referred to HEETS as cigarettes. They do this mostly out of habit and based on the assumption that others may not understand what they mean by “using IQOS”. This is a striking difference compared to e-cigarette users, who tend to strongly distinguish and distance themselves from smoking and prefer the term “vapers” [51]. A reason for this difference may be that the IQOS and HEETS provide an experience more closely related to cigarette smoking than e-cigarettes do. Although IQOS users did describe the actions of using the IQOS as different from smoking a cigarette. The reactions from bystanders to IQOS use were diverse; some users still felt stigmatized as smokers, whereas others felt more accepted as they were no longer bothering others with cigarette smoke. Acceptance by people close to them, such as a spouse or children, was highly valued by those to which it applied, and mentioned as an important advantage and reason for use.

### 4.3. Knowledge and Information Needs

Several misconceptions about IQOS came to light during our focus group discussions, such as that it does not contain nicotine or tar. It became apparent that the participants had a clear need for reliable information about the health effects of IQOS/HTPs relative to smoking. Nevertheless, most of them never made an effort to find more information on this topic. This indicates that the interest in learning more about the health effects of IQOS may not have been intrinsic, but triggered by the setting and conversation with researchers from the National Institute for Public Health and the Environment. The misperceptions and lack of knowledge about the harmfulness of IQOS may deter (prospective) users in their process of making a conscious and informed decision about initiation, continuation, or discontinuation of IQOS use. This highlights a need for further research on targeted communication strategies to facilitate the deliberation of relevant and evidence-based information about the advantages and disadvantages of HTP use. In addition, such strategies should aim to correct misperceptions about the perceived risks and benefits of HTPs.

### 4.4. Limitations

Due to the ongoing COVID-19 restrictions at the time of our study, the focus groups took place online. This may have limited spontaneous interaction to some extent. However, due to the small group sizes and active monitoring of the discussion, all participants were given the opportunity to react whenever they wished to. An advantage of our online study design is that there was less of a burden to participate; the participants could join the conversation from their own home or any other preferred location. Moreover, participants from all over the Netherlands were able to participate, since no travelling was involved. A downside is that the barrier to drop out of the study was also potentially lower. It is reasonable to assume that it is easier for participants to cancel or not show up for an online meeting than for a physical meeting at a research institute, as the latter may feel more important. This is reflected in our high attrition rate. A comparative study has shown that the content of data generated from in-person and online focus groups was similar, while online participants spoke more freely about sensitive topics [52]. Therefore, we believe that our online format did not affect the content of our discussions.

It should be noted that the group sizes in our study were fairly small and we only had one group of single users. This is due to unexpectedly low interest in participation among our target group, and high attrition, in spite of our best efforts to personally call and motivate potential participants. While the small group sizes benefited active and open discussions between all participants, they hindered a thorough and reliable comparison between the different user groups. Nevertheless, our qualitative approach ensured that the answers and information obtained from all users were included for analysis. We found that other studies did not identify additional topics to the ones discussed in our study, except for the topics of packaging [16] and gender differences [41]. These topics were the main focus of the studies describing them; thus, it is not surprising that they came up there and not in our study. The fact that other studies did not identify more topics than we did (except for the ones explicitly studied) suggests that, even with low participant numbers, we may have reached thematic saturation [53]. Moreover, because of the exploratory nature of this study, our results are not meant to convey generalizability beyond the study population and are thus restricted to a specific geographic and time context.

### 4.5. Policy Considerations

Based on our findings, several policy considerations can be formulated. First, many of our participants became acquainted with the IQOS through active sellers in stores and gas stations and through participation in panel studies. Governments that aim to discourage HTP use may therefore consider instigating bans on the active promotion and distribution of free or discounted samples of these products in stores and web shops. Moreover, they might consider restricting points of sale in order to prevent more people from becoming acquainted with HTP. This may also discourage use, as difficulty to obtain HEETS was mentioned as a disadvantage of IQOS use. Some of our participants were informed and encouraged about IQOS use in places where the promotion and advertising of HTPs were already prohibited in the Netherlands. This indicates that strict monitoring and enforcement are required to ensure such regulation is effective. Second, as many users described the IQOS in terms of stylishness and convenience, regulation of the design of HTP devices may reduce their attractiveness to (potential) users. Regulation of characterizing flavors may also reduce the attractiveness of IQOS for many users, as many are using the menthol-flavored HEETS. Some of them were prior menthol cigarette smokers who became single IQOS users, and thus successfully quit smoking cigarettes. If characterizing flavors in HEETS will be banned, these users may start to look for other menthol-flavored alternatives such as waterpipes and e-cigarettes or accessories that can provide a menthol flavor. Future research should determine how these different user trajectories influence individual and public health outcomes. Further, regulators may consider increasing taxes on HTPs to discourage use, as costs are a commonly mentioned disadvantage and reason to quit using IQOS. Finally, our participants were looking for a “healthier” alternative for smoking in the IQOS, but also were uncertain about the actual health benefit of switching. Thus, reliable information about the health effects of HTP use compared to conventional tobacco product use would aid them in making informed decisions about tobacco product use and protect their health. Such information should be provided in a way that is easy to find by the users, as they do not seem to actively carry out thorough research. For example, regulators may consider requiring this information to be provided on or accessible via the product packaging (e.g., by means of a QR code).

## 5. Conclusions

Our study explored and revealed the motivations and experiences of single-, dual-, and ex-users of HTPs. We found that almost all users started using an HTP after being encouraged by others. Single users successfully quit smoking cigarettes using the IQOS and liked the taste and experienced physical benefits. Many of them used menthol-flavored HEETS. Dual users experienced more satisfaction from smoking cigarettes and used the IQOS for specific occasions, such as social situations or places with smoking bans. Single users mainly described the advantages of IQOS use, such as health benefits, functional design, use in smoke-free places, and social acceptability, whereas dual and former users mentioned more disadvantages, including the bad taste, high costs, and the impracticalities of its use (e.g., frequent recharging). The reasons for use and the advantages and disadvantages mentioned by our Dutch users are similar to those mentioned by users in other countries. All IQOS users in our study described themselves as smokers and considered using the IQOS as an alternative way of smoking. Overall, they were not well informed about the health consequences of HTP use, but were highly interested in knowing more about them. Regulators should consider providing reliable and easily accessible information to support users in their decision-making process regarding HTP use. Furthermore, they may regulate points of sale, promotional activities, and product properties in various ways in order to reduce product attractiveness and discourage use.

## Figures and Tables

**Table 1:** Characteristics of the 19 participants per user group.

Item	Answers	Total (*n* = 19)	Single Users (*n* = 5)	Dual Users (*n* = 5)	Former Users (*n* = 9)
Gender	Male	13	4	4	5
	Female	6	1	1	4
Age in years; median (+range)		46 (26–66)	44 (30–53)	46 (30–62)	47 (26–66)
Education ^†^	Low	4	1	2	1
	Middle	4	1	-	3
	High	11	3	3	5
Frequency of use	Daily	7	3	1	3
	Multiple times a week	5	-	3	2
	Once or twice a week	4	2	1	1
	Multiple times a month	-	-	-	-
	Sporadically: less than once a month	3	-	-	3
Nr. Of HEETS per day	Less than 5	7	2	-	5
	5–10	3	1	2	-
	11–15	5	1	2	2
	16–20	2	-	1	1
	More than 20	2	1	-	1
Duration of use	Less than 6 months	7	-	1	6
	6 months up to 1 year	7	2	3	2
	More than one year	5	3	1	1
HEETS flavor used	Tobacco (e.g., Bronze, Amber, Sierra)	11	2	2	7
	Menthol (e.g., Turquoise, Green, Blue)	6	3	2	1
	Other (e.g., “sweet”)	2	-	1	1
Location of purchase (HEETS)	Online	7	2	1	4
	Gas station	1	1	-	-
	Convenience store	7	2	3	2
	Supermarket	1	-	1	-
	Tobacconist	3	-	-	3
Concurrent TRP ^1^ use on a weekly basis	None	7	4	-	3
	Cigarettes/RYO	11	-	5	6
	Cigars, cigarillos, or pipe	2	-	2	-
	E-cigarette	-	-	-	-
	Waterpipe	1	1	-	-
	Oral tobacco	-	-	-	-
	Nicotine pouches (no tobacco)	-	-	-	-
Ratio of IQOS to other TRP ^1^ use *	IQOS more often than other TRP ^1^	4	n.a.	1	3
	Equally often	2	n.a.	1	1
	Other TRP ^1^ more often than IQOS	8	n.a.	3	5
Nr. Of puffs per session	Don’t know	5	-	1	4
	1–5	2	-	1	1
	6–10	2	-	1	1
	11–14	-	-	-	-
	Until it switches off	6	3	1	2
	Differs between sessions	4	2	1	1
Do you smoke until the heat-stick is ‘finished’?	Yes	3	1	1	1
	Usually	9	3	4	2
	Usually not	4	1	-	3
	No	2	-	-	2
	I don’t know	1	-	-	1
Do you smoke two HEETS consecutively?	Yes, always	1	-	1	-
	Yes, sometimes	7	2	2	3
	No, never	11	3	2	6
Frequency of cleaning the device	After every use	2	-	2	-
	Once a day	5	-	1	4
	After 20 HEETS (as prescribed)	4	2	1	1
	Once a week	4	2	1	1
	Two-to-three times a week	-	-	-	-
	Once a month	1	1	-	-
	Never	3			3
What do you use for cleaning?	IQOS cleaning brush/set	11	4	3	4
	Own cleaning supplies	4	-	2	2
	IQOS cleaning set and own supplies	1	1	-	-
	I don’t clean	3	-	-	3

^1^ TRP: Tobacco or related products. ^†^ The categories of education are based on the definitions of the Dutch Central Bureau of Statistics (CBS), which are: Low education: this includes education at the level of primary education, vocational high school education, the first 3 years of scientific high school education, or assistant training. Middle: This includes the upper secondary education of scientific high school, basic vocational training, vocational training and middle management, and specialist training. High: This includes higher vocational education and scientific education (university). * Only applicable to dual users and ex-users. n.a: not applicable.

## Data Availability

The data presented in this study are available on request from the corresponding author.

## Supplementary Materials

The following supporting information can be downloaded at: https://www.mdpi.com/article/10.3390/toxics10060283/s1, Table S1: Interview protocol.
