# Supplementary material for: “It’s Like Jogging Next to the Highway”: A Qualitative Analysis of the Motivations and Experiences of Single-, Dual-, and Ex-Users of IQOS in The Netherlands
Source: Toxics. 2022 May 26;10(6):283. doi: 10.3390/toxics10060283 (PMC9229017; doi:10.3390/toxics10060283)
Supplement: Supplementary file 1 [file toxics-10-00283-s001.zip › toxics-1727986-supplementary.pdf]

toxics

MDPI

"It's like jogging next to the highway": A qualitative analysis of the motivations and experiences of HTP (ex-)users in the Netherlands

Anne Havermans, Lotte E. van Nierop, Charlotte G.G.M. Pauwels and Reinskje Talhout

Table S1. Interview protocol for the focus groups, translated from Dutch. Questions were formulated in past tense for former users. Prompts and further questions were only asked if the respective information did not come up in the conversation.

Topic	Participants	Questions	Prompts/further questions
1. Initiation and use	All	Can you tell me about the first time you came into contact with the product?	How did you come into contact with it?<br>What were your thoughts when you saw it for the first time?<br>Did you want to use it immediately?<br>Were you a smoker or vaper before?
		(How) did your impression change since that first time?	
		What were your considerations for using it?	Who or what played a role in your decision to use it?
		Can you tell me about the first time you used it?	Who were you with?<br>Was it your own product?<br>What did you think of it?
		What has changed since that first time?	Experience, way/frequency of use
		How did your smoking behavior/TRP use change since you started using the HTP	Smoke less/equally/more than before
	Single and dual users	Why did you continue using the product?	Is that a conscious choice?<br>In what moments/situations do you use it?<br>Are there times when you would rather not use it? When/why?
	Dual users	Why are you using both/ multiple products?	When do you use one and when the other? Why?<br>Do you use one of the two more often than the other?<br>What does the choice of product depend on?
	Dual users	Do you consider switching completely to one product?	Why/ why not?

	Single and dual users	Do you ever consider to quit using the HTP product?	Why/why not?
		What would make using HTP less attractive for you?	
	Former users	Can you tell us about when you were still a user?	When was that? Did you also use cigarettes or other products? At what times/in what situations did you use it? Were there times when you would rather not use it? When/why?
		What were your considerations for stopping to use the HTP?	Who or what played a role in your decision to quit?
		Do you ever consider to start using it again?	What would make it attractive to start again?
2. Experiences and perceptions	All	What is your general impression of the product? How do you experience using it??	What do you like about it? (advantages) What do you dislike about it (disadvantages)?
		Further questions based on provided answers, e.g.	What do you think of the device (appearance, ease of use)? What do you think of the taste (compared to cigarettes)? Do you ever use different flavors? What do you think of them? What do you think of the nicotine kick (compared to cigarettes)? What do you think of the price/cost of use? How is the use experienced by the people in your environment?
		How do you experience the use of HTP compared to smoking conventional cigarettes?	Taste, nicotine kick Do you prefer one of the products? Why?
		How do you perceive yourself, being a HTP (ex-)user?	What do you think/how do you feel about using this product? Do you consider yourself to be a smoker? Why/why not?
		What do you tell others about this product?	Do you tell others that you use this? And what do you tell them about it?

			Would you recommend it to others?<br>Are there people you would rather not tell?
3. Knowledge and information	All	Do you ever see advertising/promotion of the product?	What did you see?<br>What do you think of that?<br>What caught your eye?
		Do you ever read something about HTP/do you ever look up information?	What type of information do you search for? Why?
		What do you know about the health consequences of HTP?	How do you know/where do you get that information?<br>And what do you think about it?<br>Are you concerned about it?
Closing		Is there anything else you would like to tell us?	
		Do you have any questions about the study?	
